# Cortical and striatal circuits together encode transitions in natural behavior

**DOI:** 10.1126/sciadv.abc1173

**Published:** 2020-10-09

**Authors:** Joel Sjöbom, Martin Tamtè, Pär Halje, Ivani Brys, Per Petersson

**Affiliations:** 1 Integrative Neurophysiology and Neurotechnology, Department of Experimental Medical Sciences, Lund University, Sweden.; 2 Department of Integrative Medical Biology, Umeå University, Sweden.

## Abstract

In natural behavior, we fluidly change from one type of activity to another in a sequence of motor actions. Corticostriatal circuits are thought to have a particularly important role in the construction of action sequences, but neuronal coding of a sequential behavior consisting of different motor programs has not been investigated at the circuit level in corticostriatal networks, making the exact nature of this involvement elusive. Here, we show, by analyzing spontaneous self-grooming in rats, that neuronal modulation in motor cortex and dorsal striatum is strongly related to transitions between behaviors. Our data suggest that longer action sequences in rodent grooming behavior emerge from stepwise control of individual behavioral transitions, where future actions are encoded differently depending on current motor state. This state-dependent motor coding was found to differentiate between rare behavioral transitions and as opposed to more habitual sequencing of actions.

## Teaser

Corticostriatal circuits encode action sequencing in natural behavior.

## INTRODUCTION

Natural behaviors generally consist of distinct action phases that are sequentially implemented and that can be flexibly combined depending on the behavioral context and goals. Corticostriatal circuits are considered to play a central role in the organization of this behavior (*1*). However, rather than analyzing complex action sequences, consisting of several discrete motor programs, previous neurophysiological studies of this circuitry have mainly focused on simple movements that animals are trained to repeatedly perform in expectation of a reward, e.g., saccades or movement of a manipulandum (*2*–*4*). While this experimental design has led to several important insights, e.g., relating to the gradual automatization of learned behaviors involving bouts of repetitive stereotypic movements (*3*, *5*, *6*), it tells little about the neuronal control of natural behaviors involving complex action sequences. More recently, imaging of calcium dynamics of medium spiny neurons (MSNs) in the dorsal part of the striatum has been used to assess activity changes in this cell group in mice during spontaneous behavior in an open field (*7*–*9*). These experiments have the advantage of encompassing natural behaviors, which are, however, nonstereotypical and are displayed as a continuum of behaviors with different kinematics, including, e.g., different locomotion speeds and turning behaviors. Other studies have experimentally interfered with the corticostriatal processing by local stimulation of neuronal populations in the striatum and cortex. The outcome of these experiments have, however, been quite diverse and difficult to incorporate into a single conceptual framework, reporting changes in action selection and movement reaction time (*10*, *11*), movement velocity (*12*), contraversive turning behavior (*13*), probability of maintaining an ongoing behavior (*14*), etc. A probable limitation to this experimental approach is the patchy organization of the striatum, where cells located close to each other are considered functionally dissimilar, leading to mixed effects (*15*). Here, we instead used chronic multielectrode neuronal recordings combined with detailed analyses of complex motor behavior that allowed us to obtain information from neuronal circuits that can also belong to different, but spatially overlapping, subsystems. We analyzed the spontaneous grooming behavior displayed by rodents, which represents a natural complex behavior that includes sequential execution of discrete action phases consisting of a limited number of highly stereotypic motor programs, each with well-characterized kinematic features, which are governed by brainstem circuits (*16*). These motor programs are often implemented in specific sequences (*17*, *18*) but can also be flexibly concatenated such that the involved action phases are expressed in various combinations (see Supplementary Movies). Convincing evidence that striatal circuits are involved in the control of this behavior has been presented, e.g., by Cromwell and Berridge (*19*) who showed that lesions as small as 1 mm^3^ in the dorsolateral striatum disrupts grooming sequencing while animals are still able to execute the individual grooming phases. Using rodent grooming as a model behavior (*20*), we have, here, performed a comprehensive study of how corticostriatal networks encode the organization of actions in complex action sequencing.

## RESULTS

### Action sequencing in grooming

**Fig. 1:**
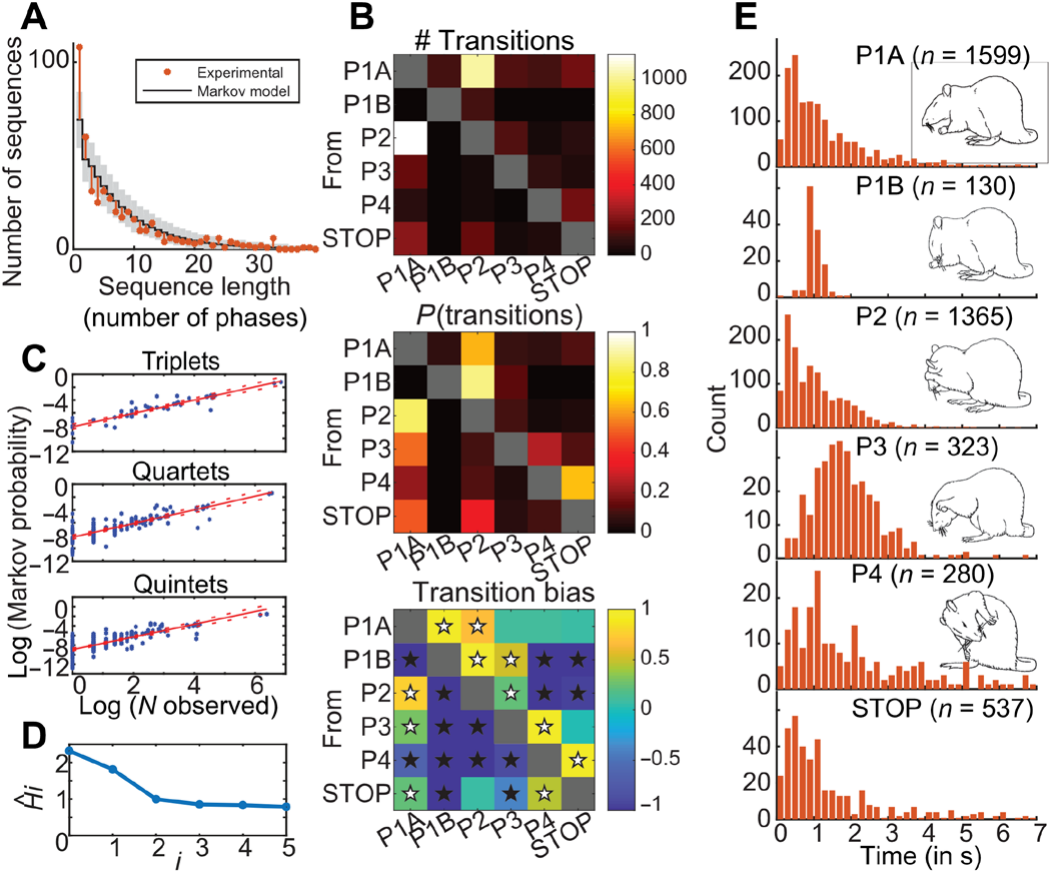
The organization of the grooming behavior. (**A**) Histogram of the number of phases making up all the observed grooming sequences (shown for sequences that have less than 40 phases, only 1.3% were longer). Markov model of predicted sequence length distribution overlaid with 95% confidence interval (CI). (**B**) Phase transitions. (Top) Total number observed of each type; range, [0 to 1144]. (Middle) Conditioned transition probability [0 to 1] (probability of going to a given column given the current state, indicated by row). (Bottom) Transition bias (transition pairs where the conditioned probability significantly deviates from the expected number on the basis of the observed frequency of the constituent parts; white/black stars denote statistical over-/underrepresentation, outside a bootstrapped 95% CI). (**C**) Calculated Markov probabilities for higher-order transition sequences containing specific combinations of phases (containing 3, 4, and 5 phases, respectively) plotted against the corresponding observed numbers within all the grooming sequences (correlation is highly significant in all cases; *P* < 0.0001; Spearman rank correlation, with *rho* = 0.92, 0.80, and 0.77, respectively). (**D**) Average conditional uncertainty in terms of Shannon information as a function of model order. Note the drop in conditional uncertainty when the previous state is known compared to when it is not (*i* = 2; first order Markov), whereas the uncertainty drops only moderately when the state two steps back is included in the model (*i* = 3). (**E**) Histograms of phase durations for the five phases and the duration of pauses between grooming bouts {STOP; behavior illustrated by inserted schematics [adapted from Aldridge and Berridge, 1998 (*21*)]}. Two-sample Kolmogorov-Smirnov test showed a statistical difference between all pairs of distributions (*P* < 0.001 after Bonferroni correction). Total number of each phase indicated by “*n*,” corresponding to (means ± SD) across recordings P1A, 42.7 ± 3.4%; P1B, 3.8 ± 1.3%; P2, 36.3 ± 4.6%; P3, 9.1 ± 2.6%; and P4, 8.1 ± 5.6%.

Spontaneous grooming was studied in seven rats and analyzed offline on the basis of video recordings. The grooming events displayed [*n* = 67 ± 32 per rat (means ± SD)] were composed of a number of discrete phases (*n* = 3697 in total), defined according to previously described criteria. These phases have been reported to often occur in a rapid succession (P1, rapid elliptical strokes; P2, asymmetrical strokes; P3, bilateral strokes; and P4, body licking], sometimes referred to as a grooming “chain” (*21*). However, in the current dataset, sequences containing several other phase combinations were much more commonly observed (see Supplementary Movies). Notably, in such “non-chain” grooming patterns (encompassing >93% of the phases), the rapid elliptical stroke phase was replaced by another motor pattern involving licking and stroking of the forepaws. This prompted us to divide the P1 phase into two different phases, where every P1B event signaled the start of an in-chain grooming event, whereas P1A was selective for non-chain events. When analyzing the total lengths of all observed sequences, we found that sequences were actually not biased toward a specific number of phases. Instead, sequence lengths followed a roughly exponential distribution, indicating that the overall probability of terminating a sequence is relatively constant and independent of the number of phases performed so far (Fig. 1A). We therefore analyzed the transition probabilities between all types of phases in further detail, including the probability of terminating a grooming sequence. The five phases differed substantially in their relative frequency of occurrence, and some phase transitions were clearly more common than others (Fig. 1B, top; while this distribution was very similar across animals and sequence length; fig. S1). Yet, transition probabilities between pairs of phases were not trivially determined by the overall frequency of occurrence of the individual phases [e.g., phase transitions that were also part of the prototypical grooming chain (1B - 2 - 3 - 4) (*18*) were found to be overrepresented; Fig. 1B, middle/bottom]. These probabilities were remarkably stable across animals with a correlation coefficient of 0.90 ± 0.063 (means ± SD; fig. S1). Similarly, the probability of exiting the grooming behavior was not the same from all phases [*P* < 0.001; chi-squared test, χ^2^(4, *n* = 3634) = 752]. We also found that the observed pairwise transition probabilities can be used to successfully model both the observed frequency of specific grooming phase permutations (Fig. 1C) and the full length of the entire grooming sequences (Fig. 1A, black line) as a series of stepwise transition probabilities (creating a mathematical Markov chain). This implies that the future state depends primarily on the current state and not on the events that occurred before it. A notion that was further strengthened by an analysis of the decrease in uncertainty about the upcoming phase as a function of model order (Fig. 1D) (*22*). Last, we found that the durations of grooming phases differed considerably both within and between different types of phases (Fig. 1E) but not between animals [two-way analysis of variance (ANOVA); *P* < 0.001 for phase type and not significant for animal]. Notably, in contrast to the other phases, 1B showed a comparatively small variance in duration and generally contained the same number of movements, i.e., six to eight rapid repetitive elliptical strokes [compare (*3*, *6*)].

### Hypothetical mechanisms underlying the sequential organization of grooming

While a probabilistic model was shown to closely reproduce the observed distributions of sequence lengths and phase sequences, it is not clear which neuronal processes could give rise to the observed transition patterns and how this activity is linked to motor commands that will ultimately give rise to the expression of the individual motor behaviors. In any case, the findings emerging from the behavioral characterizations may be taken to indicate that the neuronal control of behavioral transitions is a particularly important control mechanism for the sequential organization of grooming behavior. On the basis of this assumption, we formulated a couple of testable hypotheses concerning the neuronal motor commands that underlie the observed behavior, which extend beyond the classical view that neuronal activity in corticostriatal motor systems principally reflects the present behavior or the motor acts carried out in the very near future [see, e.g., (*23*)]. First, on the most basic level, we hypothesized that the corticostriatal system explicitly encodes the start and the end of a grooming sequence, as these events delineate transitions between distinct behavioral states. Second, we hypothesized that the involvement of the corticostriatal system in the sequential organization of grooming entails that neurons in cortical and striatal circuits specifically encode both the timing and nature of different phase transitions and that certain features of the neuronal code can account for the highly variable transition probabilities observed.

**Fig. 2:**
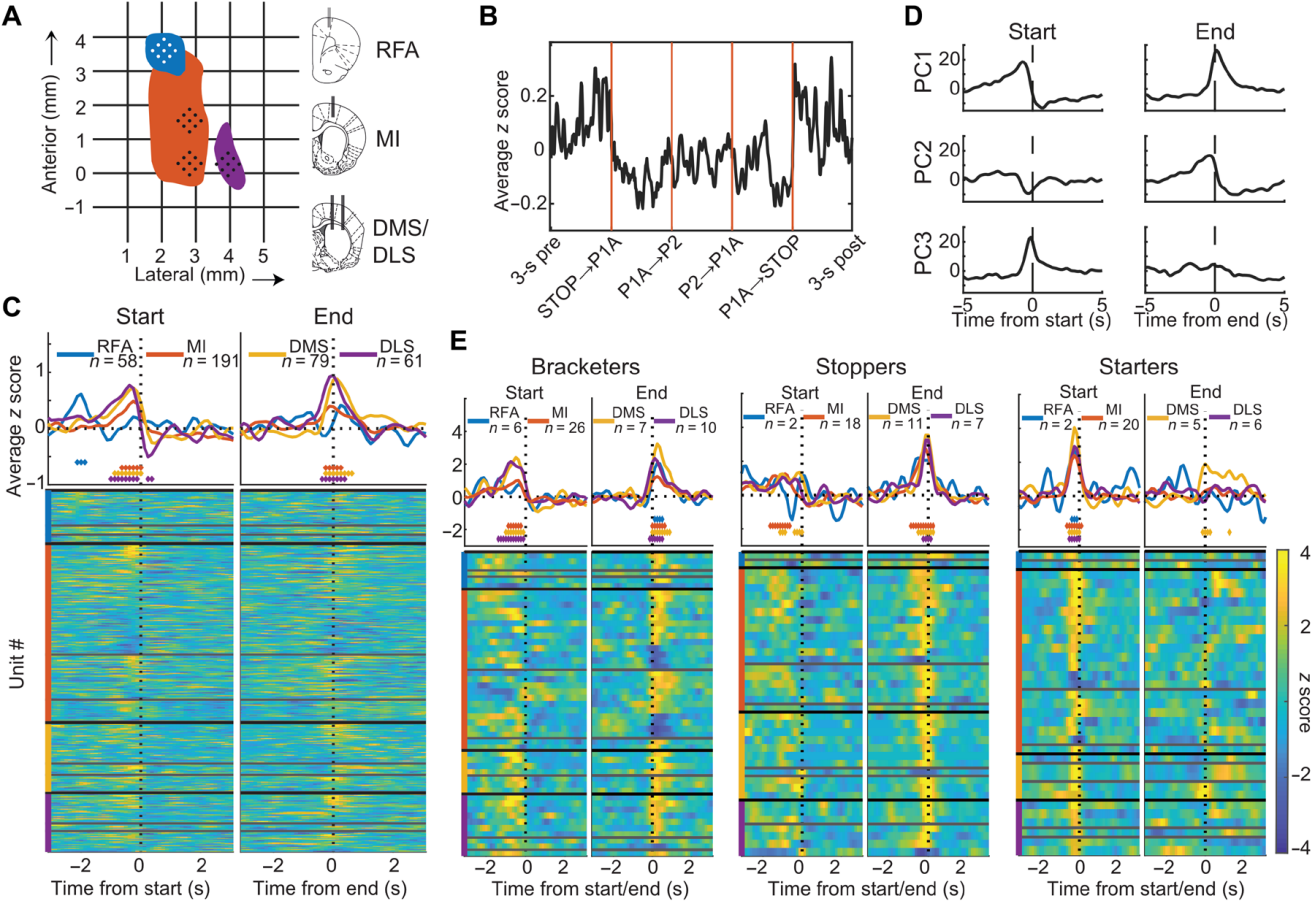
Modulation of neuronal activity in relation to the onset and end of a grooming sequence. (**A**) Recording sites are shown for the right hemisphere. Colored areas in horizontal plane indicate the forelimb representation in primary motor cortex (MI, orange), RFAs (blue), sensorimotor striatum (DLS, purple), and associative striatum (DMS) corresponds to the posterior medial group of electrodes (where DMS is located ventral to the posterior part of MI). (**B**) Average of *z-*scored firing rates of recorded units during a specific grooming sequence. The *x* axis is time warped to align transitions. (**C**) Top: Average changes in firing rate show a net increase at behavioral transitions into and out of grooming. Bottom: Perievent time histograms (PETHs) for all recorded cells, showing the average standardized rate for each cell, sorted by structure (colored vertical lines), putative cell type (PCs, INs, and unclassified, from top to bottom within each structure), and average *z* score during significant time bins. The range of *Z* is indicated by the color bar in (E). (**D**) Cell firing dynamics represented in the subspace spanned by the first three principal components [of the PETHs shown in (C)], demonstrating symmetry between transitions into and out of grooming. (**E**) Left: Cells that display significant post-end modulation (bracketers) also show pre-onset modulation. Middle: Cells that show significant pre-end modulation (Stoppers) show minimal bracketing modulation. Right: Cells that show significant pre-onset modulation without significant end modulation (Starters). Note the close resemblance in firing rate modulations between (D) and (E) and that striatum displays a predominant excitation, whereas MI modulation is more balanced, resulting in lower average firing rates. Start, first contact between forepaw and snout; end, first instance of inactivity. Diamonds in (C) and (E) denote individual time bins (125 ms) where the distribution of *z-*scored firing rates differs significantly from a standard normal distribution (*P* < 0.001; *z* test).

To test our hypotheses, we analyzed modulations of neuronal firing rates related to transitions into and out of the entire grooming sequences, as well as during the grooming behavior and in transitions between individual phases. Using chronically implanted electrode arrays, action potentials in individual neurons were recorded bilaterally in the following: primary motor cortex (MI, centered on forelimb representation), the rostral forelimb area (RFA; a supplementary motor area in rodents), and in the two striatal compartments, dorsolateral and dorsomedial striatum (DLS/DMS), which receive dense corticostriatal projections from MI and RFA, respectively (Fig. 2A) (*24*). Single-cell recordings in the cortex and striatum were attributed to originate from either externally projecting principal cells [PCs; i.e., pyramidal cells (*n* = 158) and MSNs (*n* = 80)] or from interneurons [INs; (*n* = 58/22 for cortex/striatum); fig. S2 and table S1; (*25*)].

### Encoding of grooming start and end points

When analyzing the overall changes in firing rate in the corticostriatal circuits associated with the execution of the grooming behavior, modulations of the neuronal activity was not primarily found during the expression of the active behavior. Instead, it was evident from the recordings that most of the rate modulations occurred close to the transitions into and out of the grooming behavior [as an example, the changes in average firing rate (including all recorded cells to rule out any potential effects of selection bias) associated with one of the most commonly observed grooming sequences are shown in Fig. 2B]. Overall, the firing rate of a substantial fraction of all cells (30.8%) recorded in the four structures, in both hemispheres, were found to be significantly modulated (|*Z*| > 2, with predominant rate increase) around the time a grooming sequence started and/or ended (Fig. 2C; electromyographic recordings of the forelimb muscle activity was used to confirm the time points of behavioral transitions extracted from video recordings; fig. S3). Increased activity was observed not only in MI, DLS, and DMS over about a 1-s epoch immediately before the start of the grooming (i.e., before the onset of the motor behavior) but also during a similar epoch around the time of grooming completion. These three structures all contained comparable fractions of cells showing significant modulations at onset and end of grooming sequences, although with slightly different proportions showing excitation versus inhibition (compare Fig. 2C versus fig. S4A) and were largely unaffected by the length of the pauses between successive grooming events and other sequence specific features (fig. S5). For the RFA, we observed a small average firing rate increase associated with grooming start that tended to precede the rate changes in the other three structures (peaking of ~2 s before the start). It is possible that this modulation reflects an early involvement of this premotor area in the action preparation (Fig. 2C), since no other motor behavior was consistently observed at this point in time. Thus, judging from our single-cell recordings, cortical and striatal firing rate modulations appear to be strongly associated with the state transitions into and out of the grooming behavior, rather than with the execution of the grooming behavior.

However, the neuronal control of behavior is thought to involve coordinated activity patterns in large cell populations, which may not be evident when analyzing rate changes one cell at a time. We therefore applied principal components analysis (PCA) to represent rate changes in all recorded neurons at the onset and end of grooming in a lower dimensional space. When plotting state transitions in a subspace spanned by the first three principal components, smooth trajectories with a certain symmetry between the start and end of grooming emerged, whereas the network states before and after the transitions were found to be essentially stationary (Fig. 2D). In other words, neuronal modulations are primarily associated with the switching of state per se (*26*). Moreover, the variance in standardized firing rates across neurons represented in these three dimensions implies that certain cell groups are involved in both of the transitions (as captured by PC1) and other cell groups, preferentially in one or the other, leading us to examine whether individual cells showed firing rate modulations that could be linked to these population measures.

Because modulation (|*Z*| > 2) associated with the end of the grooming sequence, in contrast to its onset, occurred both before and after the behavioral transition (compare Fig. 2, C to E, and fig. S4), the “end neurons” were split into two groups, cells that were only modulated after the end of the sequence versus others. These groups proved to be distinct in two ways. First, cells with pre-end modulation had almost no post-end modulation. Second, practically all cells that were selected for post-end modulation showed modulation also before grooming onset (Fig. 2E, left; compare Fig. 2D). These two cell groups, with cells showing either combined pre-onset and post-end modulation (“bracketers”) (*27*) on the one hand and cells showing pre-end modulation on the other (“stoppers”; Fig. 2E, middle), thus appear to serve different functions.

Similarly, by excluding all bracketers (displaying |*Z*| > 2 after the end of grooming) from the cells with a significant modulation at onset, a separate group of “starters” could also be distinguished (Fig. 2E, right; notably, the starter and stopper groups were found to be mutually exclusive). Last, in this encoding of the onset and end of grooming, INs appear to have a more prominent role than PCs in the cortex because the fraction of the cortical INs that showed significant modulations was significantly higher than that for the PCs (25/58 and 42/158, respectively; *P* = 0.020; two-proportion *z* test; see also fig. S4). Hence, this would be in line with the notion that a fluctuating release of tonic inhibition from INs is a key mediator of state transitions of this type in cortical networks (*28*).

### Encoding of phase transition events within a grooming sequence

We next investigated whether phase transitions within a grooming sequence are also associated with specific neuronal modulation patterns. All phase transitions analyzed were continuous (i.e., there were no pauses in motor behavior between the consecutive phases; see also Supplementary Movies), and the time between two consecutive transitions was often brief (48.9% of all grooming phases lasted for less than 1 s; fig. S6). Neuronal activity specifically related to phase transitions as opposed to more general in-phase behavior should, therefore, be expected to be relatively brief in time.

**Fig. 3:**
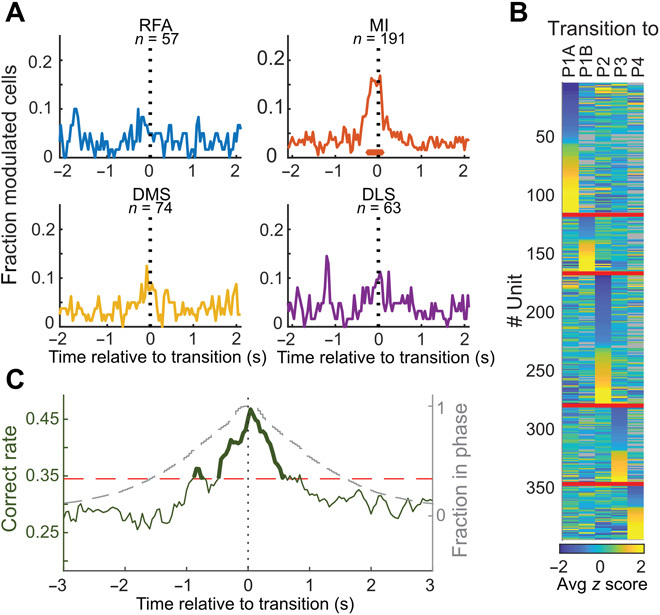
Modulation of neuronal activity in transitions between grooming phases. (**A**) Only MI showed significant modulation in association with transition events, in general (*n* = same number of PETHs, as in Fig. 2C). (**B**) Standardized firing rate modulations of all cells grouped according to preference for a specific upcoming grooming phase (transitions too rarely observed to be included are denoted in gray; means from 200 ms immediately before transition). Note that a single phase is generally preferred. (**C**) Fraction correct phase predictions for the coming phase obtained from cortical and striatal ensemble activity plotted as a function of time (dark green). Note that most information about which phase is being executed after the transition point (*t* = 0) is available in a relatively narrow time window around *t* = 0 (the much broader time interval enclosed by the gray dashed line indicates the fraction of events where no additional phase switches were observed before/after phase transition, i.e., the animal remains in the same phase pair that make up the transition at *t* = 0). Horizontal red dashed line marks chance level + 2.3 SD, corresponding to *P* = 0.01.

When aligning the neuronal activity around the time of phase transitions, the average firing rate changes were quite modest. However, we observed a small but significant reduction in cell firing rates in DMS [fig. S7; see also (*29*)]. More notably, however, a comparatively large fraction of cells in MI (15.2%) showed significant modulation (|*Z*| > 2 compared to the baseline activity), with a maximum modulation depth at the time of phase switching (Fig. 3A). Because this modulation was characterized by a reduced firing rate in some cells and an increased rate in others, the resulting net change in the firing rate at the population level was negligible. This distinct encoding of phase transition events, in general, was significantly higher in MI than in the other recorded structures (*P* = 0.018; two-proportion *z* test; 29/191 in MI versus 15/198 in the rest).

### Encoding of the nature of grooming phases in phase transitions

Averaging the over all types of phase transitions (as in Fig. 3A) could potentially mask neural events linked to one or a few specific phase transitions. We considered this issue by analyzing the modulation depth for data categorized by type of phase. First, and perhaps somewhat trivially, since it is well known that sensory feedback is an important component in corticostriatal activity (*30*), when analyzing cell activity during the execution of the five different types of grooming phases, information about the ongoing motor behavior, in terms of current phase type, was found to be encoded in the recorded structures, except for RFA (*P* ≤ 0.001, ANOVA; measured during a 200-ms window in the middle of phase execution; see also fig. S8). This activity, which probably reflects a mix of motor signal and sensory feedback, is in line with previous reports (*4*, *21*). Second, a much larger fraction of cells proved to show modulation patterns more in line with a pure motor command, i.e., specific encoding of the upcoming phase before transitioning into the new behavior, thus ruling out any role of sensory feedback [Fig. 3B; significant modulation with preference for a specific upcoming grooming phase with |*Z*| > 2 measured −200 to 0 ms before transition (i.e., a time window large enough to also precede delay time for signal transduction to spinal motor circuits and biomechanical muscle activation)], reaching a significance compared to chance level (*P* < 0.001 for MI and DLS, binomial test; table S2 and fig. S7D). To complement these single-cell analyses, we also analyzed the temporal evolution of the prediction accuracy of the upcoming phase, evaluated on the neuronal ensemble level (i.e., for a constellation of neurons recorded in parallel, using linear discriminant analysis with leave-one-out cross validation; see Materials and Methods). This analysis showed that the ensemble information builds up from about −1 s and peaks at the point of transition. After phase transition, information contents (for the ongoing phase) then relatively rapidly drops back (Fig. 3C). These results indicate that both present and future motor states are encoded in the recorded circuits (however, neuron inclusion analyses of the ensemble decoding accuracy indicated that MI might be particularly important for the encoding of the upcoming phase; see fig. S9).

**Fig. 4:**
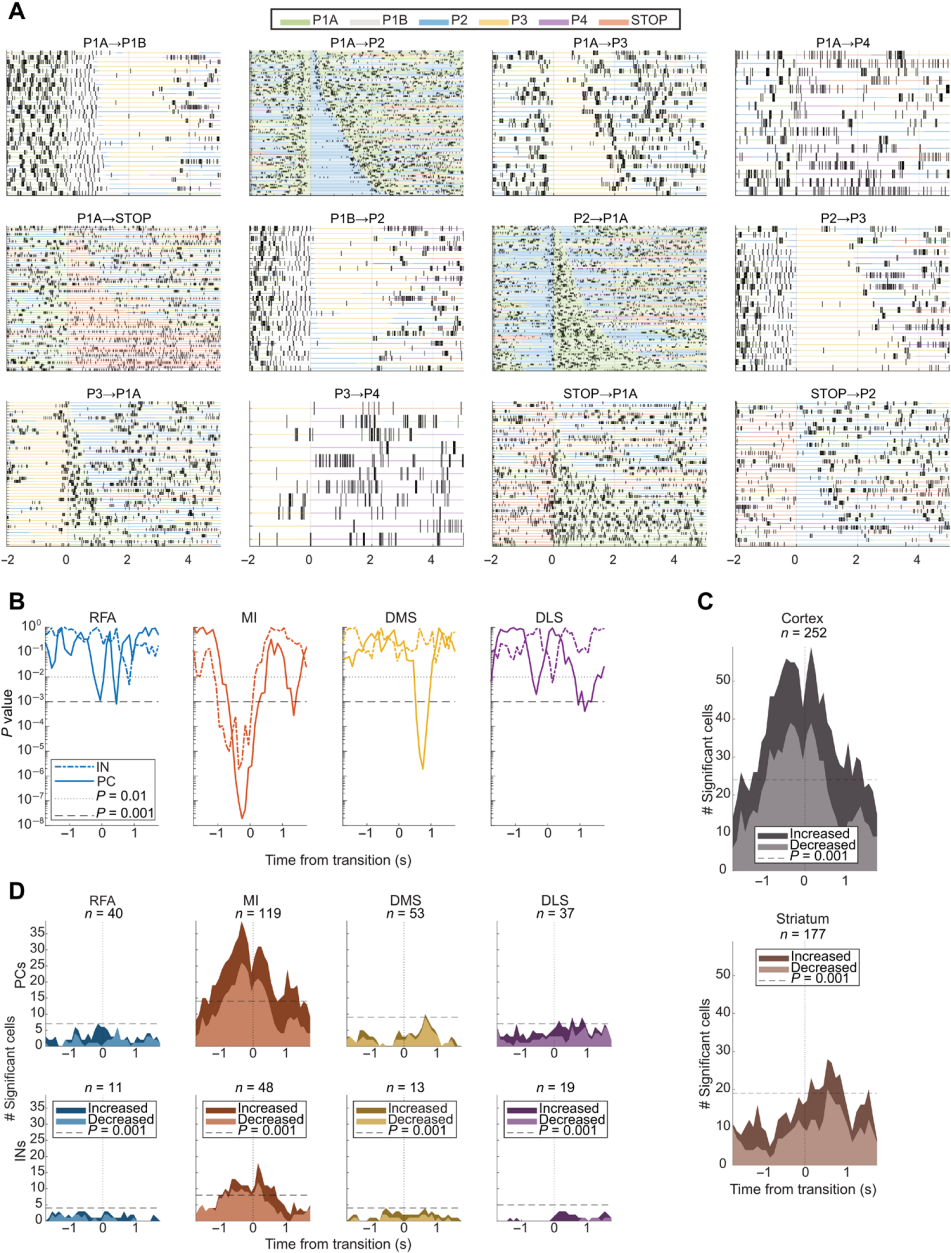
State-dependent motor commands. (**A**) Rate changes observed in an example MI principal cell in conjunction with specific phase transitions (*t* = 0 in each panel). Vertical lines mark the time points of single spikes, and colors of horizontal lines within panels denote the duration of preceding and succeeding phases. The cell displays a complex modulation pattern, showing specific firing patterns associated with present, as well as upcoming phases. Note, however, that transitions into the same phase are, in some cases, associated with diverse transition activity before transition (e.g., P1A to P2 versus P1B to P2; indicative of state-dependent motor coding). (**B**) Transition probability coding at population level. Colored lines show the *P* value for a linear model between the *z* scored firing rates and probability of transition (fitlm in MATLAB) for the respective groups. One linear model was fitted for each time bin and each group (500-ms window and 100-ms steps; see Materials and Methods). The plotted *P* values are presented without correction for multiple comparisons. (**C**) Transition probability coding for single cells. The estimated slope for each cell was compared to a shuffled CI. Cells that had a slope outside the shuffled 95% CI were considered significant (1000 shuffles of transition types). (Top) Total number of significant cells in cortex (RFA and MI) and (bottom) striatum (DMS and DLS). Light shaded area: Decreased firing rate during relatively more common transitions. Dark shaded area (not including the light area): Increased firing rate during relatively more common transitions. Dashed horizontal lines denote the number of significant cells required for a binomial test with chance level at 5% to reach alpha of 0.001 for the corresponding total *n* (without correction for multiple comparisons; fitlm in MATLAB with 500-ms window and 100-ms steps). (**D**) Same as for (C) but grouped by cell type and recording location.

A closer inspection of firing rate modulations in individual cells, which were recorded across a large number of phase transitions, hinted at an even more complex type of motor encoding, relating to the specific combination of phase pairs making up each phase transition (Fig. 4A illustrates an example cell). Specifically, rather than showing the same type of modulation patterns immediately before the transition into a specific phase, as would be expected for a generic motor command, these cells instead displayed modulation patterns that were determined by both the current and the upcoming motor state. Intuitively, this type of motor state–dependent motor commands would be well suited to create functional links between consecutive actions within longer motor sequences, similar to what has been observed in the primate supplementary motor areas (*4*) but which has not, to our knowledge, been systematically investigated in corticostriatal circuits.

While several cells appeared to display this type of differential modulation in association with different transitions, a complete examination of the encoding of all possible phase transitions was not possible, as neuronal data in this case had to be broken down on a large number of transition types, leaving relatively few events of each type during a single recording, and some phase combinations were simply too rare to be analyzed at all (as shown in Fig. 1B). Nevertheless, a more restricted analysis, searching for cells preferring a single phase transition type, was carried out (preferred phase transition versus median response for all phase transition types). This revealed a significant fraction of cells displaying a single transition type preference but only in MI (fig. S10).

### Encoding of phase transition probabilities

The role of state-dependent motor commands in action sequencing is not entirely clear, but, hypothetically, particular features of the neuronal code that differentiates between different action transitions could, e.g., explain the highly diverse transition probabilities observed for different upcoming phases within a grooming sequence, depending on the current motor state (Fig. 1B, middle). To investigate this possibility, we examined the available phase transition data (in total, 63,298 data points spread across 318 cells, distributed in the four structures and divided into putative cell types). To this end, we fitted a linear model-relating standardized firing rate, *Z*, to transition probability, *P*(*trans*), for each structure and cell type and point in time (see Materials and Methods). The model revealed a significant linear relationship for several of the analyzed groups (Fig. 4B). The observed relation between *Z* and *P*(trans) was found to display a strong temporal dependence, where the cortex showed significant correlations already before phase transition, whereas the striatum showed correlations predominantly afterward (Fig. 4B), suggesting different roles of the cortex and striatum in the control of phase transitions of different likelihood indicated by a tendency to temporally link actions pairs either forward or backward in time. In any case, this finding shows that the corticostriatal circuits encode unusual action transitions differently to more habitual transitions. We therefore next analyzed, at the single-cell level, to what extent cells belonging to the different putative groups in the different structures tended to be more active in association with low or high *P*(*trans*) events [Fig. 4, C and D; using a similar linear model, relating *Z* to *P*(*trans*), for each single cell]. As expected, single cells displayed different preferences. Nevertheless, it was evident that both MI and DMS had larger fractions of cells showing significant negative rather than positive correlation to *P*(*trans*) at time points close to the transition [(−1 s to +1 s), lighter versus darker shaded areas in Fig. 4D; MI (PCs/INs) and DMS (PCs); *P* < 0.05, Wilcoxon signed rank test with Bonferroni correction for multiple comparisons]. This implies that cells in these structures are relatively more active in association with unusual behavioral transitions rather than in the more habitual transitions.

## DISCUSSION

The central advance of this study is that it provides a first account of how neurons in cortical and striatal circuits together are involved in the encoding of action sequencing, on the spatial and temporal scale of single-unit action potentials, in a spontaneous and natural behavior involving multiple distinct motor programs (grooming phases). This study of the corticostriatal system, therefore, offers an important complementary perspective to earlier studies that have largely focused on neuronal activity recorded in a single structure, during either highly practiced rewarded behaviors [(*2*–*6*); in contrast to rodent grooming, which is a naturally developing behavior with a well-studied ontogeny (*31*)] or spontaneous behaviors expressed on a continuum of variable kinematics and movement speeds [rather than distinct stereotypic action patterns such as the rodent grooming phases; (*7*–*9*)]. Our results suggest that, in spontaneous grooming, cortical and striatal circuits have an important role in the switching of motor behavior [pointing to a role of corticostriatal network dynamics as a requirement for the system to move from one attractor state to another (*26*)] and that the full action sequences emerge from a stepwise control of the individual behavioral transitions. On the most basic level, this is manifested by the explicit encoding of the start and the end of each grooming sequence in the cortical and striatal cell groups, where net increases were most evident in the striatum (*32*). These cells were, however, found to belong to separate functional groups that we have here referred to as starters, stoppers, and bracketers, where it seems plausible that the former groups are more directly involved in direct motor control, whereas the last group (in which almost all cells with post-offset activity were found to also have pre-onset activity and consequently mark the outer boundaries of an entire action sequence) may have a role in action evaluation (*27*, *33*), as has been proposed for the matriosome/striasome cell populations (*33*, *34*). In any case, the robust encoding of behavioral boundaries in this natural behavior goes beyond the notion that on−/offset coding emerges only as a consequence of extensive overtraining of a specific sequence, as previously reported [compare (*3*, *5*)]. It is also of potential importance that previous studies have used behavioral paradigms involving the learning of either a specific number (*3*, *6*) or a specific order of actions (*5*, *32*). In the current study, analyzing natural behavior, we however found a similar type of action boundary activity in association with sequences of highly varying length and phase content.

For the switching of phases within a grooming sequence, we found that both the timing and nature of the different transitions were encoded by certain cell groups in the recorded structures. Specifically, while only MI had a significant fraction of cells, marking the time point of phase transitions independent of transition type, selective encoding of the upcoming phase type was found in both MI and DLS. Moreover, although clearly more challenging to analyze, the recorded circuits as a whole showed evidence of selective encoding of transitions involving specific combinations of phases. For a substantial fraction of cells, this translated into a negative/positive linear relationship between firing rates and the overall probability of observing a given phase transition. Thus, our neuronal data support the concept of a stepwise control mechanism in action sequencing, where individual transition probabilities are encoded by corticostriatal circuits. Such a mechanism is in complete accordance with our behavioral analyses of grooming sequences (Fig. 1, A to C), where we found that the sequence composition and length of the entire grooming sequences were well modeled by a stepwise process assuming fixed transition probabilities (a Markov chain).

These findings were unexpected in several ways and have important implications for our understanding of how motor commands are encoded in these circuits. First, it is somewhat unexpected that grooming action sequences are well described by this relatively simplistic model and that the probabilities for the different specific phase transitions were found to be almost identical across all animals (fig. S1). Second, these state-dependent transition probabilities have a direct neuronal correlate, as reflected in state-dependent motor commands, where many cells, in particular, showed a strong association between firing rate and the observed transition probability of different phase pairs. The temporal separation between the cortex and the striatum hints at the possibility that these structures link action sequences in different directions in time, forward in cortex and backward in striatum [this latter property could also be of particular relevance for action value assignment, see, e.g., (*35*)]. Although all analyses were performed, taking putative cell type into consideration, a relatively stronger involvement of the cortical INs in transitions into and out of the entire grooming sequences was the only instance where coding properties could be shown to clearly differ between cell types. This may indicate that while network activity often engages different cell types in a similar way within action sequences, INs could have a particularly important role in larger behavioral state transitions. However, at present, this remains a speculation that will need further experimental corroboration.

Last, it deserves to be mentioned that, in contrast to the encoding of the nature of phase transitions, we were unable to establish any direct links between the neuronal firing patterns and the action duration of individual grooming phases (Fig. 1C). Instead, the distributions of the phase durations for the different phases could be well described by a probabilistic drift-diffusion model [akin to previous findings analyzing spontaneous locomotion bouts in zebrafish larvae (*36*)]. Hence, to the extent that cortical and striatal circuits do, indeed, influence action duration, this neuronal control mechanism appears to be fundamentally different from the control of action sequencing and deserves to be investigated in further detail in future studies.

In the perspective of a previous work, the study having greatest resemblance to our current work is probably the study conducted by Markowitz and colleagues (*8*). A direct comparison between results is, therefore, of interest. These authors characterized neuronal modulations of individual MSNs in DLS in association with behavioral transitions in spontaneous behavior using Ca^2+^ imaging techniques and automatized behavioral classifications. They could demonstrate strong MSN modulation occurring shortly after the transition into a new behavior. While the exact temporal activation pattern in relation to behavioral transitions is a bit difficult to compare between our two studies because of the relatively slower kinetics of Ca^2+^ dynamics, our present results, nevertheless, clearly seem to corroborate their finding (see PCs in DLS in Fig. 4B).

Markowitz and colleagues also demonstrated two other properties of MSN ensembles in DLS (which was the cell group investigated in their study) that match what was, here, observed at the level of corticostriatal circuits. First, different behaviors were found to be encoded differently on the cell ensemble level (compare Fig. 3C; although the diversity of the different behaviors analyzed in terms of kinematic differences was considerably larger in their study, leading to a slightly more distinct state separation for a similar number of action classes). Second, it was reported that, out of the cells that showed different modulation for behavioral transitions of high versus low probability, a slightly higher proportion showed a negative correlation with transition probability. This is also in line with our data (corresponding to Fig. 4D, top right), while this tended to be more pronounced in DMS. However, the distinction between the medial and lateral aspects of the dorsal striatum is complicated by the lack of anatomical landmarks and may in addition not be directly comparable between mice and rats. Together, although Markowitz *et al*. (*8*) restricted their recordings to DLS, our data show that their findings, nevertheless, are largely representative of the modulation patterns observed throughout motor cortical and striatal circuits. This is of potential relevance for a large number of recent studies using similar techniques to characterize the involvement of striatal projection neurons in motor control [see, e.g., (*7*, *9*, *11*–*14*, *37*, *38*)].

An inherent limitation to our study is the need for very large datasets to compare many different combinations of actions. At the same time, because detailed manual scoring procedures were found to be required to reliably identify behavioral transitions with high temporal resolution, this inevitably constraints sample sizes. To mitigate this limitation, the current investigation contained at least four times as many cells and three times as many grooming phases as any previously published study (*17*, *21*, *39*). Furthermore, while rodent self-grooming offers several unique opportunities to investigate how complex behaviors are regulated by the brain, this behavior may not be representative for all types of action sequencing. For example, action sequencing in sensorimotor tasks typically involve a desired goal state at the sequence end, which is often represented in sensorimotor circuits in terms of desired sensory consequences. It remains unclear to what extent self-grooming has a functional goal and whether its actions are adapted by sensory information (*20*). It should also be cautioned that because electromyographic recordings were not obtained in parallel with the neuronal recordings, a direct assessment of possible links between neuronal modulations and muscle activity could not be carried out. Similarly, it is reasonable to assume that the coding principles revealed in this study are not the only control mechanism underlying action sequencing, and complementary control strategies might be involved in more complex behaviors that are not limited to stereotypic motor programs actuated by brainstem circuits (*16*). On the other hand, these situations are probably not completely distinct since more advanced goal-directed action choices are thought to act in conjunction with basal ganglia sensorimotor circuits in most everyday situations.

Together, the data presented herein provide the first description of how ensembles of neurons in cortical and striatal circuits encode the flexible sequencing of actions in a continuous, spontaneous, and natural motor behavior involving the combination of several distinct motor programs. Improving our functional understanding of the mammalian cortico-basal ganglia system is, no doubt, also an important first step toward improved therapies for motor disorders and for the development of more advanced prosthetic devices.

## MATERIALS AND METHODS

### Experimental model and subject details

Seven adult female Sprague-Dawley rats (230 to 250 g) were used in the study. The animals were kept on a natural light cycle (~12/12 hours) and received food and water ad libitum. The Malmö/Lund ethical committee of animal experiments approved all experiments in advance.

### Joint behavioral and electrophysiological recordings

Spontaneous grooming behavior was recorded during daytime hours. Before the recording started, rats were placed and habituated to a transparent rectangular 450 × 70 × 300 mm (length/width/height) box, in which they could move around freely. The relatively narrow width of the transparent box caused the rat to preferentially orient sideways to the camera, which facilitated the offline classification of the types of grooming phases performed. The behavior was recorded with a digital video camera (Teledyne Dalsa Genie HM640) triggered from an external TTL pulse generator (Master 8, AMPI) at a rate of 25 fps (frames per second), which also sent pulses to the multichannel electrophysiological recording system for the offline alignment of the behavioral and neuronal datasets. Joint recordings of electrophysiological data and grooming behavior were obtained in 14 sessions that normally lasted about 2 to 3 hours, and each lasted until the animal had performed at least 10 grooming sequences (mean of 33 and interquartile range of [21 51]).

### Classification of grooming phases

The grooming behavior was classified according to the guidelines published by Berridge and coworkers (*40*) with some minor modifications. In short, the grooming sequence was considered initiated when an animal, from a nonmoving state, first touched its snout to start grooming. For every grooming sequence, the observed action phases were categorized into one of the five previously described grooming phases, and the time of initiation and termination of each phase was identified. Videos were analyzed by two expert reviewers. The classified phase types and exact time points of transitions were cross validated between the two scorers for a large subset of the data to ensure full interscorer agreement before the full material was analyzed. Within a grooming sequence, the initiation time of a phase corresponded to the termination time of the preceding phase. A grooming sequence was considered terminated when an animal entered a period of complete immobility, for at least two video frames. The five individual phases are characterized by the following motor patterns:

1) P1A: Repetitive licking of forepaws and whiskers in vicinity to the mouth [~1 to 10 s; corresponding to the P1 out-of-chain grooming in the nomenclature of Aldridge and Berridge (*21*)].

2) P1B: Five to nine rapid and bilaterally synchronized elliptical strokes to the mystacial vibrissae [~1 s; corresponding to P1 in the nomenclature of Aldridge and Berridge (*21*)].

3) P2: Unilateral strokes over dorsal and lateral parts of the anterior half of the head (~0.5 to 2 s).

4) P3: Bilateral strokes over the posterior part of the head around the ears (~1 to 3 s).

5) P4: Postural change followed by licking of the body, generally progressing from midline outward (~1 to 30 s).

When a unilateral phase (P2 or P4) was immediately followed by the same phase on the other side, both phases were considered as one continuous phase. Moreover, because P2 was generally performed in a somewhat bimanual manner, using the contralateral paw to support the head, this behavior was not divided into left- and right-sided grooming. Last, note that the pattern of licking behavior in P4 displayed a higher degree of behavioral variability than the other highly stereotypic phases. Other types of stereotypic nongrooming motor behavior were not observed during the baseline, except for body shakes; hence, as a precaution, grooming events close to body shakes were manually excluded from the data analyzed. Note that as a result of the camera acquisition frame rate (25 fps), the temporal resolution in the time assignment of a phase transition was 40 ms (i.e., the transition was always assigned to a specific camera frame).

### Markov model of behavior

The conditional probability *P*(*Y*|*X*) of a transition to phase *Y*, given being in phase *X*, was estimated as *N*(*Y* ← *X*) / *N*(*X*), where *N*(*Y* ← *X*) is the observed number of transitions from *X* to *Y* and *N*(*X*) is the observed number of phases *X*. The transition bias was calculated as

β=log(P(Y←X)P(X)P(Y))

where the numerator is the joint probability of observing a certain transition *P*(*Y* ← *X*) = *N*(*Y* ← *X*)/Σ_*X*,*Y*_*N*(*Y* ← *X*) and the denominator is the product of the probabilities of observing each of the phases independently, *P*(*X*) = *N*(*X*)/Σ*_X_N*(*X*). Ninety percent confidence intervals (CIs) for β were calculated from the distribution created by randomly generating new values, substituting for *N*(*Y* ← *X*) and *N*(*X*), from 1000 simulated experiments using the estimated values for *P*(*Y* ← *X*) and *P*(*X*) and the same number of samples as in the experimental data.

The estimated conditional probabilities *P*(*Y*|*X*) were used to define a Markov model. A Markov chain with ~10 million transitions was generated with the MATLAB function hmmgenerate (with no hidden states). The set of grooming sequence lengths were split into ~2000 sets containing 470 sequence lengths each (equal to the number of observed lengths in the real dataset). The sets were used to calculate 95% CI. For the experimental data, 5 of 60 data points lay outside the CI. If we assume that this Markov model is the true generator of the observed sequences, then the probability of observing five or more data points outside the CI is 18% according to the binomial cumulative distribution.

### Shannon information

The estimated Shannon information, Ĥ_i_, was calculated as follows:

**Table:** 

***i***	**Ĥ**_***i***_
0	log_2_(*c*)
1	Ĥ
2	Ĥ(pairs) – Ĥ
3	Ĥ(triplets) − Ĥ(pairs)
4	Ĥ(quartets) − Ĥ(triplets)
5	Ĥ(quintets) − Ĥ(quartets)

Where *c* = 5 for the five grooming phases and

$$\hat{H}={\text{log}}_{2}N-N^{-1}\overset{c}{\underset{j=1}{\Sigma }}n_{j}{\text{log}}_{2}n_{j}$$

$$\hat{H}(\text{pairs})={\text{log}}_{2}N-N^{-1}\overset{c}{\underset{j,k=1}{\Sigma }}n_{j,k}{\text{log}}_{2}n_{j,k}$$

etc.

where *n* is the total number of each phase, pair, triplet, etc. observed, and *N* is the total sum of *n*. For *i* = 2 to 5, *N* and *n* for the lower order Ĥ were estimated from the higher order *n* [for details see (*22*)].

### Surgical procedures

All surgical procedures were carried out under antiseptic conditions. Implantations of microwire recording electrodes were performed under fentanyl/medetomidine anesthesia [0.3/0.3 mg/kg, intraperitoneally (ip)]. Electrodes were implanted bilaterally in dorsomedial/-lateral striata and in the forelimb area of the rostral and caudal motor cortex in both hemispheres targeting striatally projecting sublayers (MI, center coordinates: anteriorposterior (AP), +1.5; mediolateral (ML), ±2.8; dorsoventral (DV), −1.0 from the bregma and cortical surface; RFA, center coordinates: AP, +3.75; ML, ±2.0; DV, −1.0; and in the dorsomedial and dorsolateral striatum, respectively: AP, −0.2; ML, ±2.8; DV, −4; AP, −0.2; ML, ±3.8; DV, −3.5) (*25*). Screws in the occipital bone of the skull and anterior of the brain cavity served as connection points for the electrode ground wire, and the implant was fixated with dental acrylic attached to the screws. Anesthesia was reversed by atipamezole hydrochloride (5 mg/kg, ip) after surgery, and postoperative analgesic was administered (buprenorphine, 0.5 mg/kg, subcutaneously). The animals were allowed to recover for 1 week after implantation before the testing commenced.

### Histology

Tissue material was unfortunately not available for complete postmortem verification of electrode positions. While a more restricted analysis of the available tissue sections, in a subgroup of animals, indicated correct electrode placement, the tissue quality was regrettably of poor quality, precluding a complete three-dimensional reconstruction.

### Recording electrodes

Formvar-insulated tungsten wires (Ø of 33 μm; California Fine Wire Co.) were arranged into four 3 × 3 arrays in each hemisphere with 250-μm spacing in each dimension and cut to the length corresponding to the implantation site for each group. Each array consisted of nine recording channels and two adjacent reference channels. Reference wires were cut shorter than the recording electrodes so that they were positioned in cell-sparse regions superficial to the recording sites (cortical surface and the corpus callosum for cortical and striatal electrodes, respectively). Reference wires were deinsulated ~200 μm at the tip to lower impedance. A Ø of 200-μm silver wire was used for ground connection to skull screws. The wires were attached to a printed circuit board, linking them to board-to-board connectors (Kyocera 5602) with conducting epoxy (EPO-TEK EE 129-4), which were, in turn, connected to the acquisition system via a custom-made Kyocera-to-Omnetics connector adapter. Flexible insulated copper wires threaded through bellies of the biceps/triceps muscles were used for electromyography (EMG) recordings. A small area of the part of the wire positioned in the middle of each muscle belly was deinsulated for about 1 mm. The wires were passed under the skin to the connectors attached to the cranium.

### Signal acquisition

Local field potentials and single-unit activity data were collected and stored using a Neuralynx multichannel recording system together with the Cheetah software (v5.x). Action potentials were band-pass–filtered at 600 to 9000 Hz [low-cut 64-tap and high-cut 32-tap finite impulse response (FIR)] and digitized at 32,552 Hz. For each channel, the amplitude threshold criteria for discriminating action potentials corresponded to three SDs of the signal recorded during a 30-s period. EMG signals were band-pass–filtered at 60 to 500 Hz (64-tap FIR) and digitized at 2034.5 Hz.

### Spike sorting and cell classification

Action potentials were sorted manually offline either into single units (SUs) or multiunits (MUs) on the basis of the spike waveform features using the Offline Sorter software (Plexon Inc.). Clusters with <0.1% interspike intervals below 1.6 ms together with a separation from noise in feature space were classified as SUs. Only SUs were used in the further analysis. On the basis of the valley width, peak width, and peak-to-valley time of the average waveform, SUs were then further classified into two putative cell types per structure. The peak and valley widths were defined as the full width at half maximum. Cell type classification was performed by fuzzy *k*-means clustering with a threshold for probability of membership set to 0.75 so that SUs with a probability of membership <0.75 were unclassified (see fig. S2).

### Construction of perievent time histograms

Perievent time histograms (PETHs) of spikes from each cell were constructed by averaging over the total number of grooming events in each recording. Action potentials were divided into time bins that were adapted to fit the relative difference in transition times into and out of grooming sequences compared to between individual phases inside a sequence (125 ms for Fig. 2 and 40 ms for Fig. 3) following a temporal smoothing with a Gaussian kernel (with sigma of 167 ms for Fig. 2 and 40 ms for Fig. 3). The average firing rate of each bin was calculated and standardized using the mean firing rate and SD of all bins ±10 s around the time of the event for Fig. 2 and ±3 s for Fig. 3 (i.e., containing a mix of grooming/non-grooming behaviors in the case of Fig. 2 but only in grooming behavior for Fig. 3, after excluding periods containing the start or end of a grooming sequence).

For EMG recordings, the band-pass–filtered signals were standardized with respect to the baseline period [−4 to –1] s for each channel (six channels in biceps and three in triceps). An additional temporal smoothing (square 24-ms window) was applied before averaging the overall trials (*n* = 16) for all channels in each muscle.

### PCA of population rate changes

To represent population dynamics, PCA was applied to the full set of neurons, where each time bin in the PETH was treated as an observation and each neuron as a variable.

### Phase predictions from neuronal ensemble analyses

To estimate the information contents in neuronal ensembles as a function of time in relation to phase transitions, linear discriminant analysis was applied (fitcdiscr in MATLAB). A classifier was fitted for each time bin [including firing rates from 11 adjacent bins (±220 ms) using a Gaussian window to give increased importance to spikes close to *t* = 0] and transition on the basis of all the other transitions in that experiment and was then validated against the current phase transition (i.e., leave-one-out cross validation). The model was fitted using a pseudolinear discrimination type and uniform prior probability.

For the construction of neuron inclusion curves, multiclassifiers (fitcdiscr in MATLAB) were constructed for each recording using normalized firing rates from *n* randomly selected units from each area analyzed in a 100-ms window immediately before the phase transition. One classifier was constructed for each transition analyzed by using the firing rates from all other transitions (leave one out). To get a good estimate for the average correct rate in a subset of *n* units, this was then repeated 100 times. Subsequently, the average and SEM of the average correct rate between recordings were calculated. To estimate the chance level, the process was repeated, but all normalized firing rates were replaced with random numbers drawn from *z* scored distribution (a normal distribution with a mean of 0 and an SD of 1; using MATLAB randn).

Similar analyses were also performed, investigating the information added from each of the structures to the information contained by the total ensemble of neurons, recorded in parallel, from the other structures. In this case, correct rates were calculated using the units from all other brain structures, recorded in parallel, when including *n* units from the area analyzed.

### Quantification and statistical analysis

Statistical tests used to assess the significant group difference are specified in the main text and/or in the respective figure legends. For in-phase analysis, a 440-ms window (containing five 40-ms bins) around *t* = 0 of a PETH centered in the middle of each grooming phase was used for ANOVA. For the number of cells modulated to a single upcoming phase (|*Z*| > 2 for only one of the grooming phases), a 200-ms window right before transition was used. To test for significance, a binomial test was applied. For this, another 200-ms time window 1.5 s before the phase transition was used to calculate chance level (i.e., relatively close in time to ensure that the animal was in grooming behavior and, thus, displaying similar neuronal base-level activity). The baseline used when creating the *z* scores for the respective sample windows was a 5-s period, centered on each window (but excluding ±200 ms around the sampled window).

## SUPPLEMENTARY MATERIALS

Supplementary material for this article is available at http://advances.sciencemag.org/cgi/content/full/6/41/eabc1173/DC1

## Appendix

View/request a protocol for this paper from *Bio-protocol*.
